# Supplementary figures and images for: Protoporphyrin IX is a dual inhibitor of p53/MDM2 and p53/MDM4 interactions and induces apoptosis in B-cell chronic lymphocytic leukemia cells
Source: Cell Death Discov. 2019 Mar 11;5:77. doi: 10.1038/s41420-019-0157-7 (PMC6412042; doi:10.1038/s41420-019-0157-7)

Supplementary Figure 1

A.

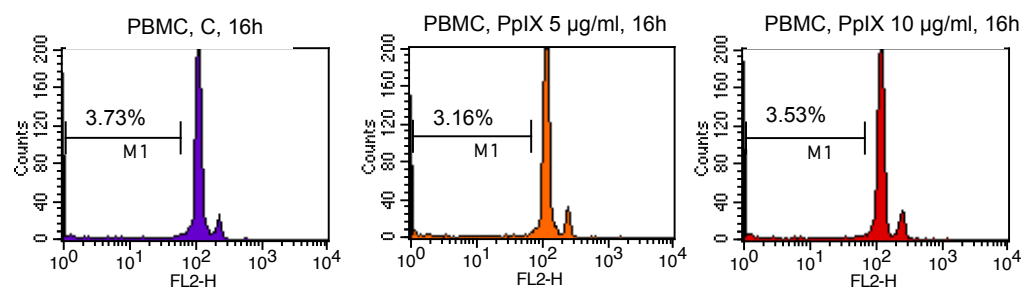

B.

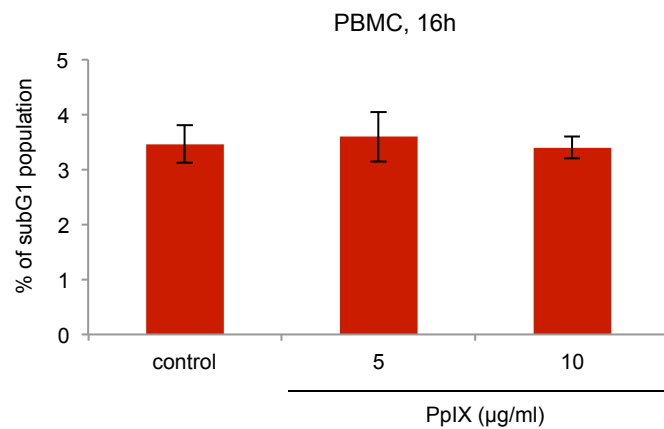

Supplement: Supplementary file 1 — Supplementary Figure 1 [file 41420_2019_157_MOESM1_ESM.pdf]
