# Supplementary material for: Protoporphyrin IX is a dual inhibitor of p53/MDM2 and p53/MDM4 interactions and induces apoptosis in B-cell chronic lymphocytic leukemia cells
Source: Cell Death Discov. 2019 Mar 11;5:77. doi: 10.1038/s41420-019-0157-7 (PMC6412042; doi:10.1038/s41420-019-0157-7)
Supplement: Supplementary file 2 — Supplementary Figure S1 [file 41420_2019_157_MOESM2_ESM.docx]

**Figure S1 related to Figure 2. PpIX does not induce apoptosis in PBMCs after 16h treatment.**

1. Histograms showing cell cycle distribution of PI-stained PBMCs treated with PpIX for 16h. C – control treated with DMSO
2. Graph showing the percentage of PBMC cells accumulated is subG1 phase after PpIX treatment for 16h. n=3, error bars represent SD values.
